# Effect of *Euphorbia hirta* plant leaf extract on immunostimulant response of *Aeromonas hydrophila* infected *Cyprinus carpio*

**DOI:** 10.7717/peerj.671

**Published:** 2014-11-13

**Authors:** Vijayakumari Pratheepa, NatarajaPillai Sukumaran

**Affiliations:** 1 Department of Aquaculture Biotechnology, Manonmaniam Sundaranar University, Sri Paramakalyani Centre for Environmental Sciences, Alwarkurchi, Tamil Nadu, India; 2 CIIMAR, Marine and Environmental Research Center, University of Porto, Rua dos Bragas, Porto, Portugal

**Keywords:** Phagocytic ratio, NBT assay, *Aeromonas hydrophila*, *Euphorbia hirta*, Immunostimulant, *Cyprinus carpio*

## Abstract

The main objective of the present study is to improve the immune power of *Cyprinus carpio* by using *Euphorbia hirta* plant leaf extract as immunostimulants. The haematological, immunological and enzymatic studies were conducted on the medicated fish infected with *Aeromonas hydrophila* pathogen. The results obtained from the haematological studies show that the RBC count, WBC count and haemoglobin content were increased in the infected fish at higher concentration of leaf extract. The feeds with leaf extract of *Euphorbia hirta* were able to stimulate the specific immune response by increasing the titre value of antibody. It was able to stimulate the antibody production only up to the 5th day, when fed with higher concentrations of (25 g and 50 g) plant leaf extract. The plant extract showed non-specific immune responses such as lysozyme activity, phagocytic ratio, NBT assay, etc. at higher concentration (50 g) and in the same concentration (50 g), the leaf extract of *Euphorbia hirta* significantly eliminated the pathogen in blood and kidney. It was observed that fish have survival percentage significantly at higher concentration (50 g) of *Euphorbia hirta*, when compared with the control. The obtained results are statistically significant at *P* < 0.05 and *P* < 0.01 levels. This research work suggests that the plant *Euphorbia hirta* has immunostimulant activity by stimulating both specific and non-specific immunity at higher concentrations.

## Introduction

The infectious diseases are a major problem in aquaculture, causing heavy loss to fish farmers. However, these diseases are becoming severe with increasing culture and in recent days, expansion of intensive aquaculture practices has led to a growing interest in understanding fish diseases. This helps the researchers for the development of medicines, which can treat or prevent the infectious diseases (Logambal, Venkatalakshmi & Michael, 2000). Antibiotics and chemotherapeutics used to control these diseases can result in the development of a drug-resistant bacteria, environmental pollution and residues in fish (Jian & Wu, 2003). It shows that extensive research is needed for the development of environment friendly and highly active moiety for the treatment.

Like humans, fish rely on both specific and non-specific mechanisms to protect themselves against invading pathogens. In fish, the primary lines of non-specific defenses are the skin and mucus, when pathogens enter into the body, cellular and humoral non-specific defences are mobilized (Dugenci, Arda & Candan, 2003). The major components of the innate immune system are macrophages, monocytes, granulocytes and humoral elements, such as lysozymes or the complement system (Secombes & Fletcher, 1992; Galina et al., 2009). The pathogen attacks the immune system in the fish and causes infectious diseases. The stimulation of the immune system of the fish can protect the fish from infectious diseases.

In recent years, increasingly more attention has been paid to the development of immunostimulants for both fish and other animals. A number of biological and synthetic compounds have been found to enhance the non-specific system in fish, which in turn protect the fish against infection caused by the pathogens (Raa et al., 1992; Jeney & Anderson, 1993; Sakai, 1999; Raa, 2000; Yin et al., 2006).

One of the major bacterial pathogens in India, *Aeromonas hydrophila*, is more abundant in water with a high organic load than in relatively unpolluted water (Yin et al., 2009). *Aeromonas hydrophila* is known to cause a variety of diseases in fish such as haemorrhagic septicaemia, infectious dropsy, tropical ulcerative disease and fin rot leading to heavy mortality in aquaculture farms (Kumar & Dey, 1988; Rath, 1993; Karunasagar et al., 1997).

In the present investigation, we were involved to find out the immunostimulant effect of *Euphorbia hirta* on the common carp (*Cyprinus carpio)* infected with *Aeromonas hydrophila*. *Euphorbia hirta* is a potential candidate and is widely used as a traditional medicinal herb in all the tropical countries. The previous studies reported that *Euphorbia hirta* extract has phenol, sugars, flavonoid, quercetine etc. as active ingredients (Sharma, Dey & Prasad, 2007; Ahmad et al., 2012). It has been utilized to treat a variety of diseases including cough, hay asthma, bowel complaints, worm infestation, kidney stones, bronchial infections as well as low milk yield (Johnson et al., 1999; Hore et al., 2006; Pratheepa & Sukumaran, 2011). This plant extract exhibited an antimicrobial activity against *Escherichia coli, Proteus vulgaris, Pseudomonas aeruginosa* and *Staphylococcus aureus* (Sudhakar et al., 2006). The ethanolic extract of *Euphorbia hirta* (EH-A001) has been found to attenuate immunologically as well as non-immunologically stimulated mast cell anaphylaxis by virtue of its potent anti-histaminic and anti-inflammatory properties (Youssouf et al., 2007). The aqueous extracts are also reported to show analgesic, anti-pyretic, anxiolytic, sedative, anti-inflammatory activities and inhibitory action on platelet aggregation (Khare, 2007).

*Cyprinus carpio* (Common carp or European carp) is a widespread freshwater fish most closely related to the common goldfish [*Carassius auratus* Linn. (Cyprinidae)] and is the number one fish of aquaculture. This fish production is affected by diseases produced by pathogens and research work in this area to improve or stimulate the immune response of this fish to fight against the pathogens (Rairakhwada et al., 2007; Pratheepa, Ramesh & Sukumaran, 2010; Harikrishnan, Nisha Rani & Balasundaram, 2003; Pratheepa & Sukumaran, 2011). Hence, the present investigation was carried out with the objective to stimulate the immune power of *Cyprinus carpio* by *Euphorbia hirta* as immunostimulant.

## Experimental

### Experimental fish collection and maintenance

*Cyprinus carpio* Linn. (Cyprinidae) used for the study was purchased from the private fish farm at Kallidaikurichi, Tamilnadu, India. Fishes of both sexes weighing about 45.9 ± 1.5 g were used for the study. They were stocked in tank (capacity 500 l) at a density of 1 g/l. In order to keep hygienic condition, the tank water was changed on alternative days and the fish were fed with balanced fish feed (Table 1). The temperature of water in fish tank was controlled between 28 and 29 °C. The water quality was also analyzed systematically at 7 day intervals to maintain optimum levels of dissolved oxygen (6.8–7.2 mg/l), pH (7.7–8.5) and ammonia (0.08–0.12 mg/l) throughout the experiment.

**Table 1:** Feed formulation used for the study.

Feed ingredients	Weight (g)	Protein content (%)
Ground nut oil cake	25.3	11.79
Fish meal	25.31	13.85
Rice bran	17.01	2.89
Tapioca	8.01	0.16
Soybean	25.3	12.01
Vitamin tablet	1	–
Cod liver oil capsule	1	–
Total	102.93	40.7

### Feed formulation

The experimental feed was prepared by mixing the selected feed ingredients (dry form) with known protein content (tested by Lowry method) accordingly to get 40.7% (Table 1). The mixture of the feed ingredients was wetted with water and steam cooked in batches and cooled. To this feed, vitamin mix and cod liver oil were added, then mixed thoroughly for even distribution. This feed mixture was pelletized through hand pelletizer until a size of 1.0 to 4 mm and dried in shade to reduce the moisture content of the feed below 10%.

### Medicated feed formulation

The medicated feed at different doses of 0, 5, 10, 20, 25 and 50 g/kg feed were formulated by adding a prescribed amount of *Euphorbia hirta* leaves extract to the pre-steam cooked and cooled feed mixture containing 40.7% protein.

### Pathogen isolation and its sensitivity analysis

The fish pathogen *Aeromonas hydrophila* was provided from the Fisheries Department, Tuticorin. The pathogen was maintained on tryptose soya agar slopes at 4 °C and was used for infecting the healthy fish. The bacterial culture was tested for its sensitivity to crude leaf aqueous extract of *Euphorbia hirta* by disc diffusion test (agar medium) using 1%, 2%, 3%, 4% and 5% concentrations of the crude extract. In consequence of this, the *Euphorbia hirta* incorporated artificial feed at different concentrations viz 0, 5, 10, 20, 25 and 50 g plant leaf extract/kg feed was given as immunostimulant to *Cyprinus carpio*.

### Experimental design

The experimental fish, *Cyprinus carpio* of uniform size (45.9 ± 1.5 g), were stocked in six troughs with ten fish each in triplicate (including control). The formulated feeds at various concentrations (0, 5, 10, 20, 25 and 50 g/kg) were given separately at 2% of body weight for an epoch of 50 days (the concentrations were fixed for the study from minimum dose). After 50 days of medicated feed feeding, all experimental fish were given only control feed. At the 50th day of immunomodulation, the fish were infected with bacterial pathogen, *Aeromonas hydrophila* through intraperitoneal injection at a dose of 1.5 × 10^4^ cells/ml. After 5 days of infection, studies were carried out once in every 5 days up to 20th day to observe the changes in haematological, immunological and biochemical responses.

### Collection of blood and antiserum

The fish were bled serially using tuberculin syringes with 24 gauge needles from caudal vein and the blood was collected in EDTA rinsed small serological tubes. The blood (without anticoagulant) collected from fish was kept overnight at 4 °C for serum separation. The serum was separated by spinning down at 3,000 rpm for 15–20 min in centrifuge. The supernatant was collected in sterile vials and the serum was kept at 57 °C in a water bath for 30 min to inactivate the complement system then stored at −20 °C for further analysis.

### Haematology

The red blood cell counts (RBC: 10^6^ mm^−3^) were determined in a 1:200 dilution of blood sample in Hayem’s solution and the white blood cell count (WBC: 10^4^ mm^−3^) from a 1:20 dilution of the blood sample in Turke’s solution with a Neubaeur haemocytometer. Haemoglobin content (Hb:gm/dl) was determined by the Cyanmethahaemoglobin method (Gowenlock, 1996). A 20 µl of the anticoagulated blood was mixed with 4 ml of Drabkin’s reagent and kept at room temperature for 4 min then read at 540 nm with UV spectrophotometer.

### Specific immune response

#### Antigen–antibody titration (Bacterial agglutination assay)

Circulating antibody titer assay was performed in 96 well microtiter plates using two fold dilutions. The titer was recorded as the highest dilution in which visible agglutination (Mat like observation) was observed. Dot like formation was considered a negative response (Vallinayagam, 1997).

### Non-specific immune response

#### Assay of phagocytic activity

The phagocytic activity assay was performed by the following modified method of Sahoo & Mukherjee (2002). Blood (100 µl) was mixed with equal quantity of bacterial suspension (1:1) in eppendorff tubes. The density of the bacterial culture was maintained throughout the experiment at 10^4^ cells/ml in PBS. The mixture was incubated for 20 min at room temperature. After incubation, a thin smear was prepared and fixed with absolute alcohol for 5 min. The smear was later stained with Giemsa stain for 5 min and the phagocytic cells that have engulfed bacteria were counted (under microscope) as positive (Seeley, Gillespie & Weeks, 1990).

The percentage of bacteria ingested phagocytes (phagocytic ratio) was calculated by the Eq. (1).

(1) \begin{eqnarray*} \displaystyle \text{Phagocytic ratio}=\frac{\text{Number of phagocytic cells with engulfed bacteria}}{\text{Number of phagocytes}}\times 100.&&\displaystyle \end{eqnarray*}

#### NBT assay

One drop of pooled (from 6 fish) heparinized blood was placed on a cover slip immediately after collection, it was placed in a humid chamber (60 mm petri dishes with a wet paper towel) and incubated for 30 min at room temperature for the neutrophils to stick on the glass. After incubation, the cover slips containing the cells were transferred upside down to a clean glass slide containing 50 µl of 0.2% filtered nitroblue tetrazolium chloride (NBT) solution and subsequently incubated for 30 min. The dark blue stained NBT–positive cells were counted under microscope (Sahoo & Mukherjee, 2002).

#### Serum lysozyme activity

Lysozyme activity was analyzed spectrophotometrically according to Sankaran & Gurnani (1972). A standard suspension of *Micrococcus lysodeikticus* was prepared in 0.066 M phosphate buffer (pH 7.0). Serum of 100 µl was added to 2 ml of bacterial suspension and was incubated at 40 °C for 20 min. After incubation, the absorbance was read at 546 nm. The lysozyme content was determined on the basis of the calibration curve and the extinction measured. Standard solutions containing 2.5, 5.0, 7.5, 10 and 12.5 µl/ml of hen egg lysozyme in 0.066 M phosphate buffer were used to develop the standard curve.

### Enzyme assay

#### Acid and alkaline phosphatase

Acid and alkaline phosphatase activity was estimated by the following method (Pattabiraman, 1998). Serum sample (10 µl) was mixed with 5 ml of substrate solution and the absorbance was read at 420 nm immediately. Then the substrate solution with serum sample was incubated at 37 °C for 30 min and the absorbance was read at 420 nm. The optical density (OD) difference was noted and the phosphatase activity was calculated by using the Eq. (2).

(2) \begin{eqnarray*} \displaystyle \text{Phosphatase activity (}\mathrm{\mu} \text{M PNP/mg protein)}&&\displaystyle \nonumber\\ \displaystyle \quad =\frac{\text{OD difference of test}}{\text{OD of standard }{X}_{1}\text{/sample taken }{X}_{1}\text{/incubation time }{X}_{1}\text{/mg protein}}\times \text{conc. of standard}.&&\displaystyle \end{eqnarray*}

#### Serum peroxidase

Serum peroxidase was analyzed by the procedure of Murugesan & Rajakumari (2005). To a 1.4 ml of amino antipyrine, 1.5 ml of H_2_O_2_ was added and to this mixture, 0.1 ml of serum sample was added and the extinction was measured at 510 nm for about 5 min. The solution without serum sample was served as blank and the standard solutions containing different concentrations of peroxidase in 0.066 M phosphate buffer were used to construct a standard curve. The results are expressed in IU/ml.

### Pathogen clearance

#### Pathogen clearance in blood

Pathogen count in blood sample was carried out by the following procedure of Vallinayagam (1997). The blood sample was serially diluted and the pathogen were counted by using pour plate technique with specific agar (thiosulfate citrate bile salts sucrose (TCBS) agar, Himedia) in the petriplates and then incubated at 37 °C for 24 h. The values were expressed in cfu/ml.

#### Pathogen clearance in Kidney

Kidney of infected fish weighing about 0.1 g was homogenized and then centrifuged for 10 min. The centrifuged solution was subjected to serial dilution. The diluents were appropriately pour plated with nutrient agar and incubated at 37 °C for 24 h. The values are expressed in CFU/0.1 gm.

### Disease resistance

The effect of plant leaf extract incorporated feed for the disease resistance (survival percentage) on fish (*n* = 20/group) were determined. The fish were artificially challenged with dose of 1.5 × 10^4^ cells/ml of live virulent pathogen, *Aeromonas hydrophila.* The mortality was observed for 20 days and the average of triplicate set was used to express in terms of percentage of survival.

### Statistical analysis

The data collected were statistically analyzed using two way analysis of variance (ANOVA) to test the effects of experimental feed for all parameters. Student’s *t*-test was used to test differences among individual means and the control. The difference was recorded as significant when *P* < 0.01 and *P* < 0.05.

## Results

The immunomodulation study of the leaf extract of *Euphorbia hirta* on the freshwater fish *Cyprinus carpio* (infected with *Aeromonas hydrophila*) has provided significant results. The haematological study on the fish shows that the maximum RBC count of 2.63 ± 0.034 × 10^6^ cells/mm^3^ was noticed on the fish fed with 25 g leaf extract incorporated feed. After the 5th day of infection with the pathogen, the RBC count was decreased in all the fish and the least RBC count was noticed in fish fed with the control feed. The RBC count was increased after the 20th day of infection with the highest RBC count of 1.92 ± 0.02 × 10^6^ cells/mm^3^ at 25 g leaf extract/kg incorporated feed.

The fish showed high haemoglobin content of 10.38 ± 0.04 g/dl on feed having 50 g leaf extract of *Euphorbia hirta*/kg and the control fish showed 8.75 ± 0.08 g/dl. The haemoglobin content was decreased after infection and on the 15th day onwards the haemoglobin content was increased in all the fish. The maximum haemoglobin content of 7.21 ± 0.02 g/dl was noticed for the fish fed with 25 g leaf extract of *Euphorbia hirta*/kg.

The maximum total WBC count of 56.80 ± 0.87 × 10^4^ cells/mm^3^ was obtained from the fish fed with 50 g leaf extract of *Euphorbia hirta*. After the 10th day of infection, the peak WBC count of 67.60 ± 2.82 was observed on the fish fed with 50 g leaf extract of *Euphorbia hirta*/kg feed and the WBC count was decreased (23.94 ± 2.24 × 10^4^cells/mm^3^) in the control fish on the same day. Among the different concentrations tested, the fish fed with 50 g extract feed markedly enhanced the WBC count.

The log_2_ antibody titer results show that the antibody production has increased as the concentration of leaf extract increases and the maximum log_2_ antibody titer of 10.67 ± 0.58 was obtained with 50 g leaf extract (Table 2). The *Euphorbia hirta* were able to increase the antibody production only for 5 days after infection and in further days the antibody production was decreased in all the fish fed with *Euphorbia hirta* leaf extract.

**Table 2:** Effect of different concentrations of leaf extract of *Euphorbia hirta* on antigen antibody titration in *Cyprinus carpio* infected with the bacterial pathogen, *Aeromonas hydrophila*.

Concentrations of leaf extract (g/kg feed)	Post challenge of pathogen (Days)			
	5	10	15	20	*t*-value
0	3.33 ± 0.58	3.67 ± 0.58	2.67 ± 0.58	1.67 ± 0.58	–
5	6.00 ± 0.00	5.67 ± 0.57	5.00 ± 0.00	4.33 ± 0.57	4.20^**^
10	7.33 ± 0.58	6.33 ± 0.58	5.67 ± 0.58	5.00 ± 0.00	4.89^**^
20	9.00 ± 0.00	8.33 ± 0.58	7.00 ± 0.00	6.33 ± 0.58	6.43^**^
25	10.33 ± 0.58	10.00 ± 0.00	8.33 ± 0.58	7.67 ± 0.58	8.01^**^
50	10.67 ± 0.58	10.00 ± 0.00	9.33 ± 0.58	8.67 ± 0.58	11.09^**^
*t* value	–	0.29^NS^	0.96^NS^	1.40^NS^	–

**Notes.**

Each value is the mean of three individual observations with a standard deviation.

** *P* < 0.01.

NS Not significant.

**Figure 1:**
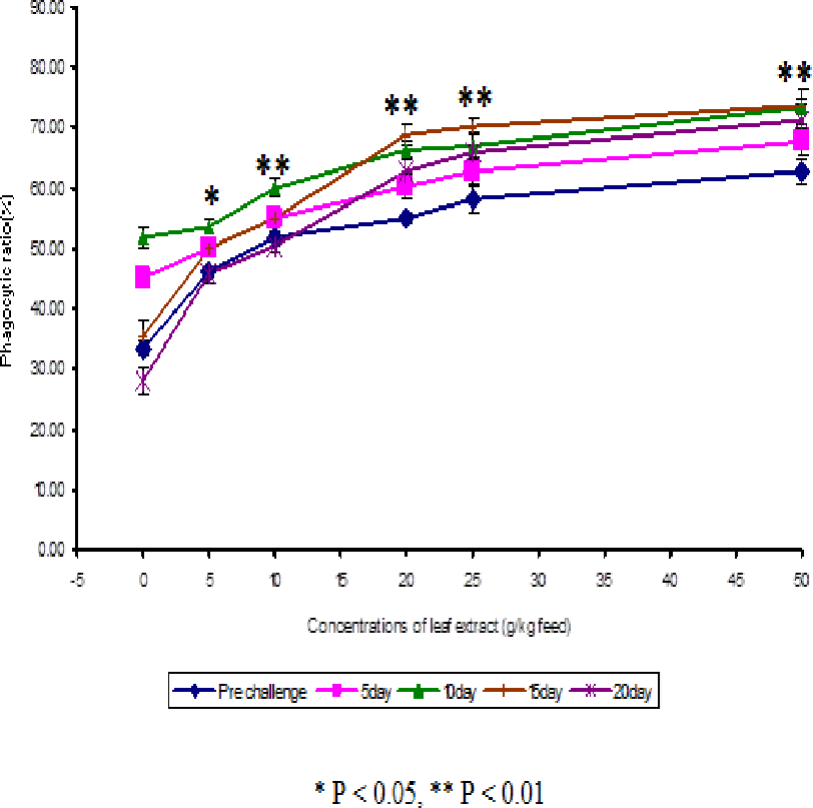
Effect of different concentrations of leaf extract of *Euphorbia hirta* on number of NBT positive cells in *Cyprinus carpio* infected with the bacterial pathogen, *Aeromonas hydrophila*.

The results obtained from the study of phagocytic activity of *Euphorbia hirta* on *Cyprinus carpio* is graphically represented in Fig. 1. From the Fig. 1, it is clear that the fish fed with higher concentrations of leaf extract of *Euphorbia hirta* were able to increase the phagocytic ratio and the maximum phagocytic ratio of 62.67 ± 2.05 % was noticed on the fish fed with 50 g leaf extract of *Euphorbia hirta*/kg feed. The peak phagocytic ratio of 73.67 ± 1.65% was found on the 15th day after infection with the feed having 50 g leaf extract. In control fish, the phagocytic ratio was increased up to 10 days after infection and in further days it was decreased.

**Figure 2:**
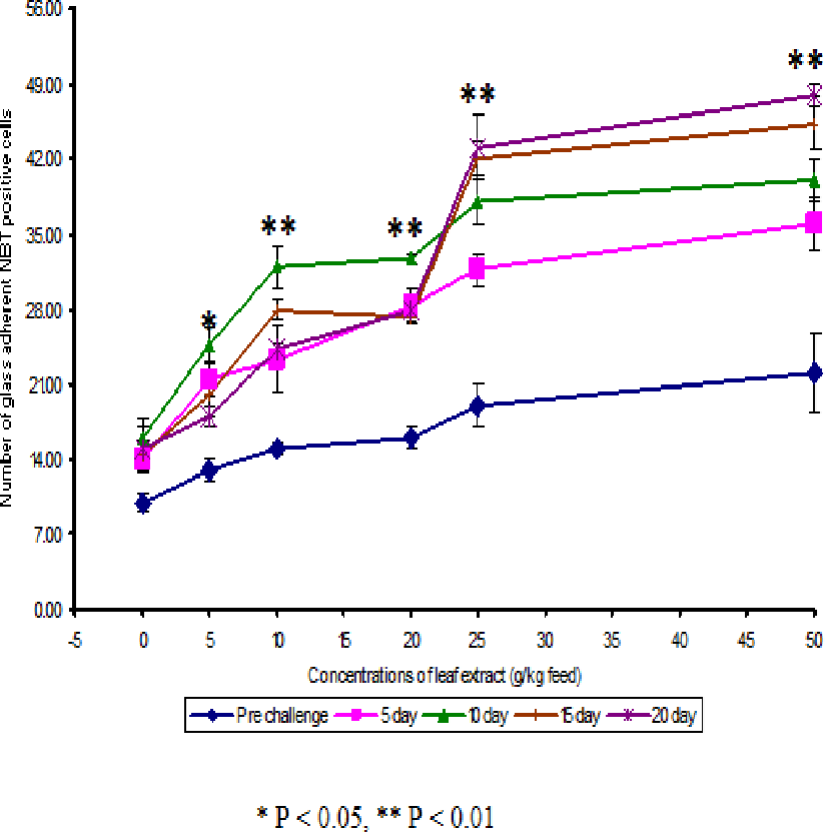
Effect of different concentrations of leaf extract of *Euphorbia hirta* on phagocytic ratio (%) in *Cyprinus carpio* infected with the bacterial pathogen, *Aeromonas hydrophila*.

The NBT assay results show that the glass adherent NBT positive cells was increased as the concentrations of the leaf extract were increased and the number of positive cells were found to be 13.00 ± 1.00, 15.00 ± 0.58, 16.00 ± 1.00, 19.00 ± 2.00 and 22 ± 3.61 for the fish fed with 5, 10, 20, 25 and 50 g leaf extract of *Euphorbia hirta*/kg feed respectively (Fig. 2). After infection with the pathogen, the number of NBT positive cells was increased for about 10 days in control and in medicated fish. The fish fed with 50 g and 25 g leaf extract concentraction were able to increase the number of NBT positive cells upto 20 days and the values were found to be 48.00 ± 1.00 and 43.00 ± 3.00 respectively.

The stimulated serum lysozyme activity was obtained on the fish fed with higher concentrations of leaf extract. The serum lysozyme activity was maximum on the 10th day after infection in all fish including the control and the values were found to be 7.58 ± 0.29, 8.33 ± 0.21, 8.90 ± 0.13, 10.76 ± 0.35, 13.88 ± 0.19 and 15.61 ± 0.30 µg/ml for the fish consumed 0, 5, 10, 20, 25 and 50 g leaf extract of *Euphorbia hirta* respectively. After 10th day of infection, the serum lysozyme activity was decreased and the least serum lysozyme activity of 4.55 ± 0.241 µg/ml was observed in the control fish on the 20th day after infection.

The serum acid phosphatase activity of 0.501 ± 0.01 (IU/l) was found on the control fish after the 5th day of infection and subsequently, the values were decreased up to the 20th day. On the 10th day after infection, the maximum serum phosphatase activity of 1.912 ± 0.07 (IU/l) was observed for the fish fed with 25 g leaf extract and the value of 1.199 ± 0.181 (IU/l) was obtained on the 20th day after infection with the same dose. The maximum serum alkaline phosphatase activity of 1.003 ± 0.10 (IU/l) was noticed at 25 g leaf extract. The activity was enhanced up to 10 days after infection and the value of 0.601 ± 0.01 (IU/l) was noticed for the control fish on the 10th day after infection.

In control fish, the serum peroxidase activity was found to be 21.16 ± 2.00 units/ml and after infection with the pathogen, the serum peroxidase activity was increased. The maximum peroxidase activity of 28.27 ± 2.32 units/ml was noticed on the 10th day after infection. The fish fed with 50 g leaf extract yielded maximum serum peroxidase activity of 37.83 ± 1.83. After infection with the pathogen, the serum peroxidase activity was increased up to 10 days with the maximum of 45.65 ± 2.42 units/ml on 25 g leaf extract of *Euphorbia hirta/*kg feed.

**Figure 3:**
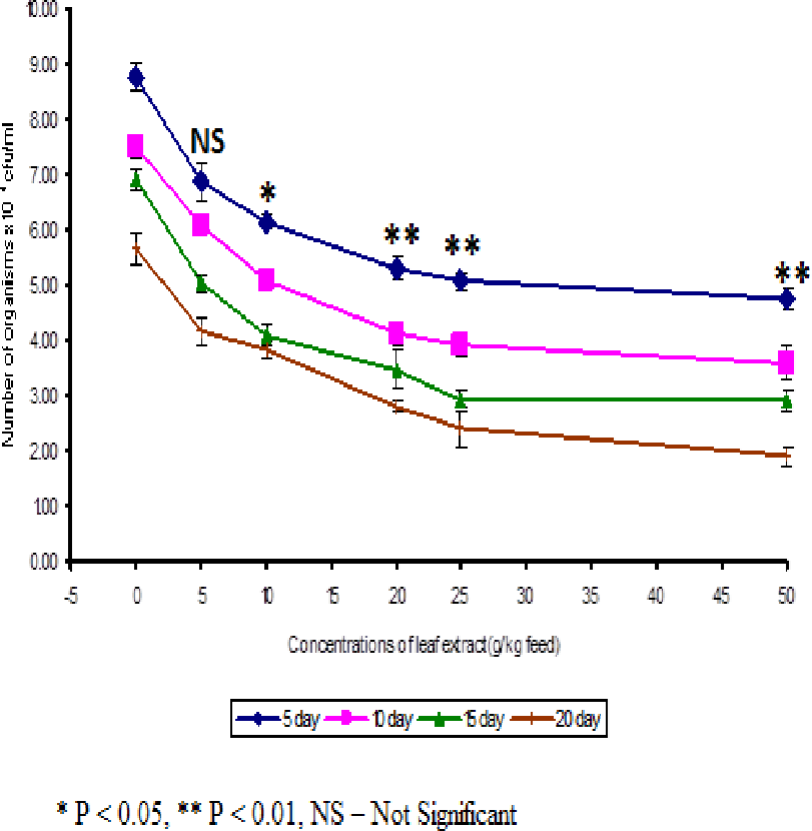
Effect of different concentrations of leaf extract of *Euphorbia hirta* on pathogen clearance in the blood of *Cyprinus carpio* infected with the bacterial pathogen, *Aeromonas hydrophila*.

The least blood pathogen count of 4.73 ± 0.21 was noticed on the fish fed with 50 g leaf extract of *Euphorbia hirta* on the 5th day after infection (Fig. 3).

**Figure 4:**
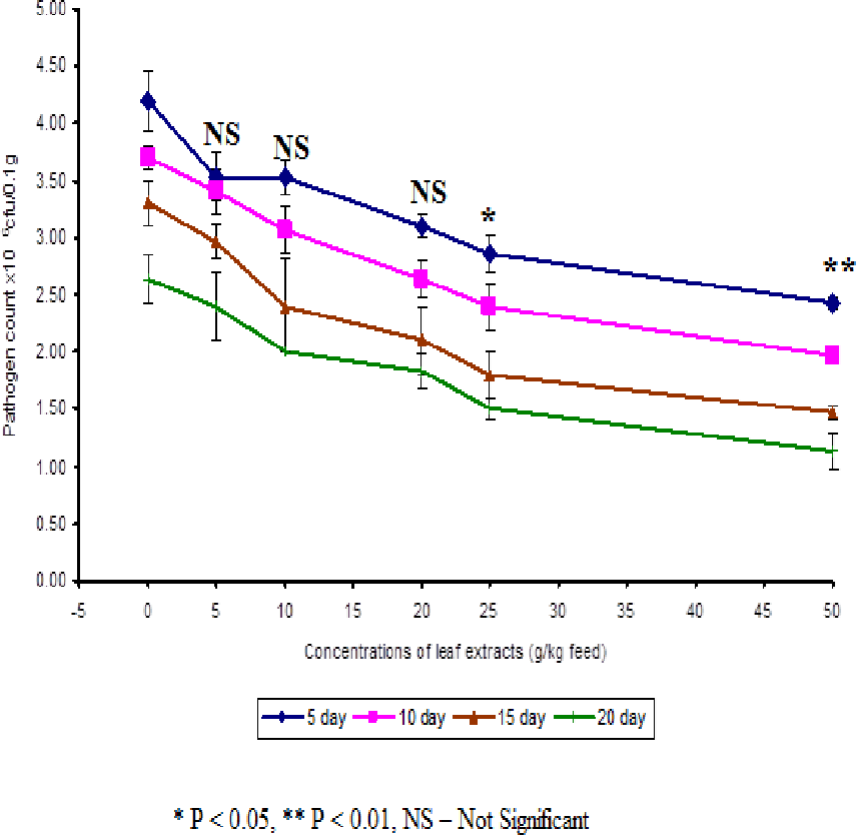
Effect of different concentrations of leaf extract of *Euphorbia hirta* on pathogen clearance in the kidney of *Cyprinus carpio* infected with the bacterial pathogen, *Aeromonas hydrophila*.

In control fish, the pathogen count of 8.73 ± 0.25 × 10^4^ cfu/ml was noticed on the 5th day after infection. The results described that the fish fed with 50 g leaf extract was able to clear the pathogen from the blood effectively than other concentrations. The fish fed with control feed showed 4.20 ± 0.26 × 10^6^ cfu/0.1 g of pathogen in the kidney on the 5th day after infection. Among the medicated fish, 50 g leaf extract of *Euphorbia hirta* showed 2.43 ± 0.06 × 10^6^ cfu/0.1 g of pathogen in the kidney (Fig. 4).

The fish fed with 50 g leaf extract of *Euphorbia hirta* showed better elimination of the pathogen till 20 days after infection. Aqueous leaf extract of *Euphorbia hirta* when supplemented in feed were effective (*P* < 0.05 level) in eliminating the pathogen at higher concentrations (25 and 50 g) of leaf extract and the leaf extract was able to eliminate the pathogen during the10th and 20th day after infection. All the concentrations of leaf extract of *Euphorbia hirta* enhanced the survival percentage significantly at lower concentration (5 g) (Table 3).

**Table 3:** Survival percentage of *Cyprinus carpio* fed with feed incorporated with leaf extract of *Euphorbia hirta* and infected with the bacterial pathogen, *Aeromonas hydrophila*.

Concentrations of leaf extract (g/kg feed)	*E. hirta*
**0**	61.67 ± 2.89
**5**	71.67 ± 2.89^*^
**10**	73.33 ± 2.89^**^
**20**	80.00 ± 0.00^**^
**25**	86.67 ± 2.89^**^
**50**	90.00 ± 0.00^**^

**Notes.**

Each value is the mean of three individual observations with a standard deviation.

* *P* < 0.05.

** *P* < 0.01.

## Discussion

The haematological study results reveal that the fish fed with feeds having the leaf extract of *Euphorbia hirta* significantly enhanced the RBC count, haemoglobin content and WBC count when compared to that of the control fish. It is also observed that the RBC count, haemoglobin content and WBC count were maximum in fish fed with feed having 50 g leaf extract of *Euphorbia hirta.* The previous study of Cooper, Welster & Harris (1963) reported that mitochondria play a significant role in iron metabolism in developing erythrocytes. *Euphorbia hirta* induced erythropoiesis and lymphopoiesis increased the RBC count, haemoglobin content and WBC count. Kumar et al. (2006) in their experiments found that RBC count and haemoglobin content were significantly reduced due to bacterial challenge, but dietary starch (gelatinized and non-gelatinized) had no effect on it, whereas the dietary starch enhanced WBC count. The haematological results of the present study reveal that the leaf extract was able to reduce the immunosuppression caused by the pathogen through increasing the haematological response.

Although all the concentrations of leaf extract of plants enhanced antibody response, the higher concentrations (25 to 50 g) of *Euphorbia hirta* were only able to stimulate higher antibody production, but statistically no clear concentration dependency in the enhancement of antibody production was noticed (Table 2). The present findings are in agreement with the earlier studies, which reveals that a significantly increase in haemagglutination antibody titers was observed from *Phyllanthus emblica* in *Cirrhinus mrigala* (Regina Mercy, 2006). Chand et al. (2006) found that giant freshwater prawn *Macrobrachium rosenbergii* (de Man) fed with bovine lactoferrin showed significant increase in agglutination titers. The result from the present study also reveals that the leaf extract in the feed of carp was able to stimulate specific antibodies against the challenged bacteria and rise humoral immunity as observed in the previous studies.

In this present investigation, several assays were carried out to test the efficacy of leaf extract as immunostimulant so that an accurate index of immune competence could be assessed.

The present study clearly elicits that the plant leaf extract was able to stimulate the phagocytic activity and enhanced the phagocytic ratio significantly. Phagocytic activity is mediated by cytokines such as macrophage activating factor secreted by peritoneal lymphocytes (Graham & Secombes, 1988). The results of the present study show that chemotaxis process was stimulated by the higher concentrations (25 and 50 g) of *Euphorbia hirta* (Fig. 1).

Some researchers have reported that there was a significant increase in phagocytic activity observed in 0.5% KM-110 (Korean mistletoe-110) group compared with the 0.1% group (*p* < 0.05). It indicates that 0.5% KM-110 concentration is suitable for stimulating maximum phagocytic activity resulting in a high amount of reactive oxygen intermediates (ROI) production (Choi et al., 2008). The results also reveal that macrophage migration in the presence of exo antigen enhanced with the incorporation of different concentrations of leaf extract of *Euphorbia hirta*.

The NBT-positive cells were found to be significantly increased (*P* < 0.01) with increase in days after infection on fish. This is probably due to the increase in lysozyme activity. Lysozyme production was mainly based on the neutrophils and monocytes present in the blood. This is also reported by the fact that lysozyme activity in fish fed with feeds having leaf extract was found to be higher than the control, suggesting that the production of more number of NBT positive cells in the fish.

The leaf extract of *Euphorbia hirta* administered through feed enhanced the non-specific defense mechanism in terms of increased number of activated neutrophils. The previous findings supported the view that the external stimulants like plant extracts stimulated the activity of NBT positive cells in the blood of fish, as evidenced by the results of present investigation in common carp (Fig. 2). This is supported by the fact that serum lysozyme and phagacytic ratio also enhanced in fish fed with feeds having leaf extract.

The lysozyme activity has been found to be modulated by a range of factors including stress, water temperature, infection of foreign materials, nutrients etc. (Fletcher & White, 1973; Sakai, 1999). The increased levels of lysozyme in fish fed with feeds having plant leaf extract could be the result of an increment in the number of phagocytes (macrophage) which secrete more amount of lysozyme. The higher concentrations (25 to 50 g) of *Euphorbia hirta* significantly enhanced the serum lysozyme activity. It is also reported that the medicinal plants are effective in enhancing serum lysozyme activity. When *Labeo rohita* was fed with feeds having extract of *Achyranthes aspera* (Rao et al., 2006) and n-PUFA (Misra et al., 2006b), the serum lysozyme activity was increased considerably. Poly herbal formulation (Immunoplus) (Kumari, Sahoo & Giri, 2007) and *Magnifera indica* (Sahu et al., 2007) in the feed of *Labeo rohita* also enhanced the serum lysozyme activity. All the above studies show the effect of herbal plants on lysozyme activity, which agree with the present study by the enhanced serum lysozyme activity.

Phosphatase enzyme is considered a member of lyzosomal enzyme and is widely considered a valuable parameter of macrophage activation (Sveinbjornsson & Seljelid, 1994). The results of the acid and alkaline phosphatase activities indicate that the fish, *Cyprinus carpio* fed with feed having leaf extract of *Euphorbia hirta* showed significant enhancement in the phosphatase activity when compared with the control fish. The enhancement of serum phosphatase activity in fish may caused by the increased production of enzyme by the macrophage cells. Dalmo & Seljelid (1995) reported that the lipopolysaccharide (LPS) stimulated the macrophage cells for the higher enhancement of acid phosphatase, when compared to the control macrophage cells. Rao et al. (2006) reported that *Achyranthes aspera* enhanced the serum alkaline phosphatase activity in *Labeo rohita*, as observed in common carp in the present study.

The azurophilic granules of neutrophils release myeloperoxide enzyme during oxidative respiratory burst activity, which is measured through the serum peroxidase activity. From the results obtained in the present study, it is clear that the leaf extract at different concentrations were able to stimulate the serum peroxidase activity as evidenced from the increased enzyme activity in fish fed with feeds incorporated with leaf extract of the plant, when compared with control fish. Esteban et al. (2005) noticed that serum peroxidase had no significant difference when sea bream was fed with lactoferin. Whereas in the present study, the enzyme activity peaked up to 10 days after infection and moderate effect was observed beyond 50 days after feeding with the medicated feed.

The results of the pathogen clearance elicit that the fish consumed feeds with leaf extract were able to eliminate the pathogen from the blood and kidney significantly (Figs. 3 and 4). The leaf extract was able to eliminate the pathogens on the 10th day after bacterial challenge. This is supported by the results of specific and non-specific immune parameters. The decrease in specific and non-specific immune response after the 10th day may be due to the elimination of pathogen from the body of fish. Yoshida et al. (1993) noticed that bacterial counts in the blood and spleen decreased in the fish, *Clarius garipinus* treated with glucan. *Acalypha indica* leaf extract in the feed of tilapia was also able to eliminate the pathogen from the kidney and blood (Sukumaran & Anita, 2001). Sahu et al. (2007) and Misra et al. (2006a) observed that increased pathogen clearance was observed in rohu when the fish was fed with *Magnifera indica* and Tyfstin incorporated feeds respectively. They have suggested that the total immunostimulant property of the plant extracts may be the reason for better pathogen clearance, as indicated by total specific immune responses.

Results of the present investigation on disease resistance (survival) revealed that the incorporation of leaf extract of *Euphorbia hirta* in feed protects the fish (increased protection), *Cyprinus carpio* against the pathogen, *Aeromonas hydrophila* (Table 3). The mechanism by which the survival was augmented appears to be positively correlated with increased phagocytosis, neutrophil activity, lysozyme activity, etc. Among the different concentrations tested, the groups fed continuously with feed having higher concentrations (25 and 50 g) of *Euphorbia hirta* resulted in maximum protection. Earlier studies revealed that dietary supplementation of *Ocimum sanctum* leaf extract enhanced disease resistance against *Aeromonas hydrophila* in *Oreochromis mossambicus* (Logambal, Venkatalakshmi & Michael, 2000). Misra et al. (2006a) reported that multiple injection of tuftsin might have maintained the activation of phagocytic cells for a long period, which in turn led to long-term protection in the fish. Following challenge with *A. hydrophila* less survivability was observed in the control group (56.65%) than the group fed the experimental feeds. The group fed 0.5 g Euglena kg^−1^ dry diet showed the highest percentage survival (75%) (Das, Pradhan & Sahu, 2009). Sahu et al. (2007) observed that mango kernel stimulated the immunity and made *Labeo rohita* more resistant to *Aeromonas hydrophila* infection as observed in common carp in the present study.

## Conclusion

In general, the immunostimulant (plant extract) was found to stimulate antibody response, lysozyme and phagocytosis and other immunological function in fish at higher concentrations. The pathogen clearance study elicits that the leaf extract was able to eliminate the pathogen from its circulatory system in *Cyprinus carpio*. The disease resistance study indicates that the fish fed with plant leaf extract was able to increase their survival percentage significantly. This study also reveals that the scope of using extract of *Euphorbia hirta* as an immunoprophylatic in the health management in culture of carps. Finally appropriate field trials are necessary before using the aqueous extract as immunoprophylatics to prevent infectious diseases in fin fish aquaculture.
